# PD-L1 Expression Correlated with p53 Expression in Pediatric Glioblastoma Multiforme

**DOI:** 10.3390/brainsci11020262

**Published:** 2021-02-19

**Authors:** Jakub Litak, Wiesława Grajkowska, Justyna Szumiło, Paweł Krukow, Ryszard Maciejewski, Jacek Roliński, Cezary Grochowski

**Affiliations:** 1 Department of Immunology, Medical University of Lublin, Chodźki 4a, 20-093 Lublin, Poland; jakub.litak@gmail.com; 2 Department of Pathology, The Children’s Memorial Health Institute, Aleja Dzieci Polskich 20, 04-730 Warsaw, Poland; W.Grajkowska@ipczd.pl; 3 Chair and Department of Clinical Pathomorphology, Medical University of Lublin, 20-093 Lublin, Poland; justynaszumilo@umlub.pl; 4 Department of Clinical Neuropsychiatry, Medical University of Lublin, 20-439 Lublin, Poland; pawelkrukow@umlub.pl; 5 Department of Anatomy, Medical University of Lublin, 20-400 Lublin, Poland; maciejewski.r@gmail.com; 6 Laboratory of Virtual Man, Chair of Anatomy, Medical University of Lublin, 20-093 Lublin, Poland

**Keywords:** pediatric glioblastoma multiforme, PD-L1, GBM

## Abstract

High-grade gliomas are infrequent in the pediatric population compared to adults, nevertheless, mortality and morbidity caused by malignant gliomas in this group of patients remain significant. PD-L1 and PD-1 Immune checkpoints (IC) molecules maintain immunological balance between activation and suppression. Eighteen patients with a histopathological diagnosis of pediatric glioblastoma multiforme (GBM, WHO IV) were studied. In total, PD-L1 expression was detected in 8 patients (44%). The molecular aspect of IC and immunotherapy targeted on PD-1/PD-L1 axis in pediatric population may be a promising adjuvant therapy in pediatric glioblastoma multiform treatment, however, this subject requires further investigation.

## 1. Introduction

High-grade gliomas are infrequent in the pediatric population compared to adults, nevertheless, mortality and morbidity caused by malignant gliomas in this group of patients remain significant. The incidence of high-grade gliomas is 10%. Moreover, GBM incidence varies between 3 and 15% of all primary central nervous system tumors. Despite treatment, long-term outcomes are generally poor. The five year survival time for pediatric patients is ca. 20% [1]. Glioblastoma Multiforme patients older than 3 years of age are treated with surgery, adjuvant chemo and radiotherapy. In younger patients’ radiotherapy should be delayed to avoid significant adverse effects affecting the developing brain tissue. Optimal therapeutic strategy is still uncertain [2]. Current studies point at molecular aspects of the underlying neoplastic processes as the target of modern therapies [3,4].

Immune checkpoints (IC) PD-L1 and PD-1 maintain the immunological balance between activation and suppression. The PD-1/PD-L1 axis controls the processes of negative selection concerning lymphocytes, becoming auto reactive in secondary and primary lymphoid organs. PD-1/PD-L1 knock-out animal models present aggressive autoimmunity. GBM cells self-induce PD-L1 expression by multi receptor activation such as IFNGR, IFNAR, EGFR, TLR. PD-L1 expression in GBM activates subunits of PD-1 on the microglia surface and suppress T cell response [5].

PD-1/PD-L1 ligation activates the SHP-1/SHP-2 complex and recruits it to interact with ITSM. ITSM is an intercellular tail attached to transmembrane region of PD-1. This process promotes ZAP 70, PKC, and CD3 dephosphorylation. In parallel, the Ras -MEK-ERK and PI3K-Akt pathways are also inactivated. These disturbances globally inhibit the anti-tumor response and thus encourages the invasion of glioblastoma [6]. Overexpressed PD-L1 in GBM cells induces the apoptosis of T cells and activates T-reg cells in close microenvironment, promoting the immune escape of GBM. Relevant research works indicate the GBM expression of PD-L1 as a tumor biomarker [7]. The successful blockage of immune checkpoints in melanoma adult patients has been underlined in several studies. The promising results of IC inhibition bring new insight in oncology of brain tumors [8]. The effectiveness of Pembrolizumab, Duravalumab, Nivolumab, Ipilimumab, and Pidilizumab, members of PD-L1/PD-1 inhibitors, have been thoroughly analyzed in ongoing trials [9,10,11,12].

TP53 is the most frequently deregulated gene in carcinogenesis. The modified product of TP53 transcription and translation, the loss of p53 has been implicated in migration, the evasion of apoptosis, proliferation, and invasion of glioblastoma cells. The expression of p53 correlates with unfavorable outcomes, highlighting its crucial role in glioblastoma therapy. Olig2 expression determines oligodendroglial differentiation and plays crucial role as a prognostic factor in glioma classification.

Ki 67 is a nuclear protein associated with cell cycle and proliferation. As a non-his- tone protein connected with ribosomal RNA and transcription processes, it is a sensitive marker of differentiating cells [13,14,15,16].

The presented study evaluates the PD-L1 level in 18 pediatric GBM pediatric patients. To our best knowledge, this is the first study analyzing the expression of PD-L1 in such a number of patients with a homogenous histopathological diagnosis of glioblastoma multiforme (WHO G IV). Moreover, a correlation between PD-1 occurrence with p53, Olig2 and Ki 67 expression is performed in order to find significant molecular interplay in pediatric glioma tissue.

## 2. Methods

### 2.1. Subjects

Eighteen patients with a histopathological diagnosis of pediatric Glioblastoma Multi- forme (GBM, WHO IV) were randomly selected for the study from the Children’s Me- morial Health Institute database in Warsaw, Poland, from 2014 to 2019. Clinical and histopathological data were collected on the basis of medical documentation (Table 1, Table 2, Table 3 and Table 4). The histopathological diagnosis of GBM was performed according to the WHO guidelines by a board-certified neuropathologist (W.G.). An age below 18 as well as the diagnosis of GBM were the only inclusion criteria. A board-certified pathologist (J.S.) performed all tumor scoring for PD-L1. This research was approved by the local medical ethics committee of the Medical University of Lublin (KE-0254/330/2019) and was carried out in compliance with national legislation and the Declaration of Helsinki.

### 2.2. Tissue Histology and Collection Process

The tissue was collected during standard tumor resection. The samples were pre- served using 4% phosphate buffered formaldehyde and paraffin-embedded with standard procedures.

H&E staining was performed on 4 μm paraffin sections using standard protocols. Immunohistochemistry was applied using an autostainer (Dako) after heat-induced epitope retrieval in a citrate buffer.

### 2.3. Immunohistochemistry

Formalin-fixed paraffin-embedded tumors of pediatric glioblastoma were cut using a microtome into a 4 μm slices, which were placed on a Thermo Scientific™ SuperFrost Plus Slides for a better tissue adhesion in order to perform immunohistochemistry (IHC). The analysis was performed on a Dako Omnis IHC platform (GI100, Santa Clara, CA, USA). Selected reagents were used in the IHC analysis: PD-L1 IHC 22C3 pharmDx staining set (GE006) including monoclonal PD-L1 antibody 22C3 clone (Dako Omnis, Santa Clara, CA, USA), negative control (Dako Omnis, Santa Clara, CA, USA), high pH detective sys- tem EnVision Flex Mini Kit (Dako Omnis, Santa Clara, CA, USA, GV823), wash buffer (20x) (GC807, Dako Omnis, Santa Clara, CA, USA). In order to remove the paraffin, a low pH Envision Flex Target Retrieval Solution (Dako Omnis, Santa Clara, CA, USA) was used. Tonsil tissue was used as a positive control and Negative Control Reagent was used for the negative control.

PD-L1 was assessed using PD-L1 IHC 22C3 pharmDx staining protocol and for the negative control PD-L1 IHC 22C3 pharmDx Negative Control Reagent staining protocol.

### 2.4. Statistical Analysis

Quantitative characteristics of the group were presented as means and standard deviations (M, SD) for parametric variables and as percentages and frequencies for non-parametric values.

The planned correlations between biomarkers such as PD-L1, p53, Olig2, Ki 67, due to non-parametric type of variables, were computed with an application of Spearman’s R coefficient. To verify possible differences between subgroups selected on the basis of criteria such as sex (male/female), hemisphere affected by neoplasm (right/left), and su- pratentorial versus subtentorial location of pathology, the non-parametric Mann–Whitney U test was used due to the small number of observations in these subgroups and the ordinal level of analyzed variables. For all statistic tests, the threshold of significance was set at p 0.05, adjusted for Bonferroni correction in case of multiple testing.

## 3. Results

### 3.1. Subsection

The number of GBM pediatric patients enrolled in the study was 18. The mean age at final diagnosis day was 10, 74 y.0. (SD = 5.05), ranged (min. 4 months, max. 17 years). Six female and 12 male patients (33% vs. 66%) were included. All the patients had the same diagnosis—Glioblastoma Multiforme (G IV). The study cohort received surgical intervention, and 17 out of 18 individuals underwent adjuvant treatment (Table 1 and Table 2).

All the patients had a single lesion. Fourteen lesions were presented in the supratentorial location (77%) versus 4 in subtentorial cases (33%). The lesion within the left hemisphere was observed in 10 cases (55%) versus 4 in right (22%); additionally, 4 patients presented midline infiltration (22%) (Table 3).

### 3.2. PD-L1 Status

**Figure 1:**
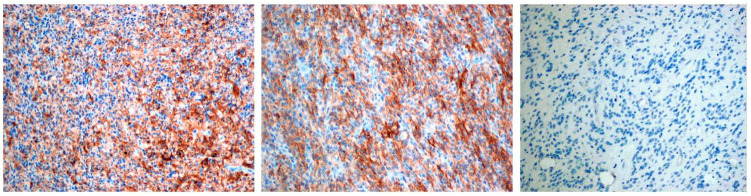
The first two pictures present positive PD-L1 immunostaining in pGBM cells, the last picture presents negative PD-L1 immunostaining. Objective magnification ×10 for each photo.

PD-L1 staining was detected mainly in glioblastoma cells and was graded in a 4-stage intensity scale 0 none; 1 weak <20%; 2 moderate 20–40%; 3 high >40%. In total, PD-L1 expression was detected in 8 patients (44%): 5 cases with mild expression, 1 case with moderate, and 2 with high levels of expression (Figure 1, Table 3).

### 3.3. Molecular Status

All tumor samples were investigated for GFAP, Olig2, Ki 67, p53 and Synaptophysin expression. Scale 0-none 1-present. Ki 67 was evaluated in a 4-grade expression scale 0-none 1-weak 2-moderate 3-high. GFAP expression was detected in 4 cases (22%). The occurrence of Olig 2 protein was found in 4 out of 18 cases (22%). Thirteen patients (72%) presented Ki 67 expression, 2 patients with weak and 11 with high level of expression. p53 was detected in 9 patients. Only one patient was detected as a case with synaptophysin expression (Table 4).

The hypothesis of PD-L1 correlation with chosen molecular patterns was put for- ward. Our consideration focused mainly on p53, Olig2, and Ki 67. A positive relationship between PD-L1 and Olig2 R = 0.61, *p* < 0.01 was found and was determined to be statistically significant. The same results were presented by PD-L1 and p53 correlation R = 057, *p* < 0.05. No statistically significant correlation was found between PD-L1 and Ki 67 pair R = 0.38 *p* > 0.05 (Table 5)**.**

We also investigated the differences concerning biological parameters such as location in two subgroups supratentorial and subtentorial location. The lack of statistically significant differences (all *p* > 0.05) was found in this analysis. The same results were obtained concerning the comparison (Left/Right) subgroups (all *p* > 0.05).

Additionally, there were no differences in range of occurrence of biological parameters such as gender and age (all *p* > 0.05, Mann–Whitney U test).

To sum up, our study revealed PD-L1 statistically significant correlations with p53 and Olig2 protein in pediatric population of GBM patients. The lack of a significant relationship with Ki67 was confirmed. Biological aspects such as sex, location, and left/right brain hemisphere had no significance.

## 4. Discussion

The presented study revealed that PD-L1 expression is present in pediatric glioblastoma multiforme patients. Different levels of expression oscillated around 45% in 8 out 18 cases. There are few reports in the literature concerning Pd-L1 expression in children with malignant glioma. A Similar observation was made by Allison et al. [17] This study demonstrated that half of 14 cases cohort diagnosed with LGG presented PD-L1 expression [17]. Majzner et al. [18] identified PD-L1 in 6 out of 20 cases with pediatric high-grade glioma [18]. Interestingly, the intensity of PD-L1 occurrence corresponds with an effective response to ICIs related pathway [19]. A meta-analysis performed by Hao et al. [20] analyzed 9 studies of adult PD-L1 positive glioblastoma patients [20], which suggested the high incidence of PD-L1 to be connected with poor patient survival. No differences were found between PD-L1 and gender as well as age, which was similar to results in this study. This relation was also observed in non-small cell lung cancer (NSCLC) treated with pembrolizumab, where the expression intensity was over 50% and PD-L1 expression was linked with better treatment response [21]. Another study concerning PD-L1 occurrence in adult glioblastoma patient indicated a higher level of expression at 88%. The analyzed cohort was significantly larger, had contained 117 cases. These findings clearly confirm experimental models based on the hypothesis of PD-1/P PD-L1 axis have a prominent role in forming favorable microenvironment for gliomatous infiltration [22]. Al Harbi et al. [23] presented results of nivolumab, PD-1/PD-L1 axis inhibitor in pediatric glioblastoma patient resulting in a 60% reduction of GBM infiltration, improvement in clinical status, and continuous 10-month durable response [23]. Moreover, the study performed by Gorsi et al. [24] indicated better results of Nivolumab treatment in patients with a higher rate of PD-L1 comparing to those without expression. Interestingly, the progression free survival (PFS) in PD-L1 positive group versus PD-L1 negative was 13.7 weeks vs. 4.2 weeks (*p* = 0.08) [24].

The p53 protein, called the “Genome Guardian”, functions as a regulator of transcription, promotor of mitotic cycle arrest, integrator of stress signals, and initiator of apoptosis. Additionally, it has protective properties concerning genome integrity. The mutation of TP53 is connected with glioma progression. The p53 protein is associated with more proliferative, less apoptotic, and more invasive phenotype of primary GBM. The deregulation in p53 signaling pathway is one of the most commonly found epigenetic disorders with incidence around 84% in adult population [25,26]. Our study demonstrates 50% occurrence rate of p53 in IHC analysis, which is similar to the results presented by Suri et al., who demonstrated p53 expression level oscillating ca. 63% in a group of 30 glioblastoma pediatric cases [27]. The differences between adult and pediatric GBM were explained by Watanabe et al. [28]. Their study indicated lower p53 accumulation in primary GBM affecting commonly pediatric patients compared to secondary being more popular among adults (25% vs. 65%) [28]. Pollack et al [29] had similar results concerning p53 expression oscillating ca. 58% of all pediatric glioblastomas. Moreover, the level of p53 expression was correlated with PFS rate (5 years) in cohort of 74 pediatric patients. Interestingly, the group with p53 low expression had higher PFS at 5 year than group with p53 over-expression (44 ± 6% vs. 17 ± 6%) (*p* = 0.001) [29].

Olig2 protein is commonly promoted in adult gliomas controlling oligodendrocytes development and expressed mainly in LGG. Pediatric glioma tumors present different genomic profile. Pediatric glioblastomas have the highest ratio of Olig2 occurrence, which are Ki-67 positive (mean 16.3%). Otero et al. presented results concerning Olig2 expression in 90 patients of different brain tumors. Among them, only 4 were diagnosed with GBM (WHO G IV) characterized with the highest Mean Olig2 score. Our study indicates a 22% expression of Olig2 in glioblastoma multiforme cohort. In GBM Olig2 seems to be prominent GSC indicator and Olig2 expression was associated with Ki67 occurrence. Moreover, 88% of Ki 67 positive cells were Olig2 positive in pediatric glioblastoma [30]. This study indicated PD-L1/Olig2 correlation as a statistically significant (PD-L1/Olig2 R = 0.61 *p* <0.01) and this relation suggests polymorphous character of GBM tissue, how- ever, it requires further investigation.

Ki 67 levels in glioblastomas are relatively high comparing to other brain cancers. Its expression is active throughout all phases of the cancer cell cycle excluding the resting phase. Several studies have tried to evaluate the level of expression with uncertain outcomes. The study performed by Alkhaibary et al. [31] presented the results excluding a significant correlation of Ki67 expression and overall survival in adult patient [32]. In Sharma et al.’s [31] study, Ki67 expression among GBM pediatric patients was relatively higher compared to those another brain tumor. They also found no significant correlation between K67 rate and outcome [31]. Our results suggest lack of significant correlation between PD-L1 and Ki67 (PD-L1/Ki 67 R = 0.36 *p* > 0.05).

This study investigated significant correlation between PD-L1 and p53 expression in the pediatric GBM population (PD-L1/p53 R = 0.38 *p* < 0.05). These biological molecules have been proven to have unfavorable prognostic value. The synergistic effect of both could strengthen unsuccessful outcome. Cortez et al. [33] propose a novel mechanism of p53 interplay with PD-L1 expression through miRNA-34. This short miRNA molecule promotes the immune evasion of glioblastoma [33]. Some relations concerning PD-L1 and p53 expression were investigated in 976 adult Glioblastoma samples confirming our results. Further investigation on glioblastoma pediatric cohorts is required [34]. Clinical trials concerning IC inhibitors such as antiPD-1/PD-L1 agents bring a new hope for pediatric population suffering from glioblastoma multiforme. Despite uncertain results with pembrolizumab, in heterogeneous groups of patients with gbm, including small cohorts of pediatric patients, further immunological and epigenetic profiling is required to minimize the impact of these efficacy-reducing factors [35,36].

Only the precise molecular screening of treated cohort and meticulous investigation of biological mechanisms can optimize qualification for adequate treatment [37,38,39,40,41,42,43,44,45].

## 5. Conclusions

The analysis of the pediatric cohort presented in this study brings interesting results. PD- L1 correlation with p53 expression could be an interesting area to look for prognostic properties of both. The molecular aspect of IC and immunotherapy targeted on PD-1/PD-L1 axis in pediatric population may be a promising adjuvant therapy in pediatric glioblastoma multiform treatment, however, this subject requires further investigation in further studies.

## Figures and Tables

**Table 1:** Selected demographical and clinical data of patients enrolled to the study.

Mean Age 10, 74 Years (min. 4/12; max. 17)	
Characteristic	No. (%)
Gander	
Male	12 (66%)
Female	6 (33%)
Primary/Secondary	
Primary	18 (100%)
Secondary	0 (100%)
Surgical Treatment	
Total resection	10 (55%)
Subtotal	5 (28%)
Biopsy	3 (17%)
Adjuvant treatment	
Yes	17 (94%)
No	1 (6%)

**Table 2:** Summary of patients’ characteristics.

Location	No. (%)
Left temporal lobe	*n* = 5 27%
Left frontal lobe	*n* = 4 22%
Brainstem	*n* = 2 11%
Cerebellum Vermis	*n* = 2 11%
Left occipital lobe	*n* = 1 5%
Right parietal lobe	*n* = 1 5%
Right thalamus	*n* = 1 5%
Right frontal lobe	*n* = 1 5%
Right temporal lobe	*n* = 1 5%

**Table 3:** Location of Glioblastoma Multiforme (GBM) infiltration among the GBM pediatric cohort (*N* = 18).

Subject No	Age (Years)		Gender	Histological Diagnosis	Tumor Location	Ki67 (%)	PD-L1 (%)
**1**	6	M	Glioblastoma Multiforme	Left temporal lobe		Negative	Negative
**2**	11	M	Glioblastoma Multiforme	Left frontal lobe		Negative	Negative
**3**	9	F	Glioblastoma Multiforme	Left temporal lobe		28	Positive (5)
**4**	14	F	Glioblastoma Multiforme	Left occipital lobe		34	Negative
**5**	17	F	Glioblastoma Multiforme	Right frontal lobe		8	Negative
**6**	5	M	Glioblastoma Multiforme	Pons		26	Negative
**7**	6	M	Glioblastoma Multiforme	Left frontal lobe		32	Positive (50)
**8**	14	M	Glioblastoma Multiforme	Right parietal lobe		50	Positive (5)
**9**	8	M	Glioblastoma Multiforme	Left temporal lobe		Negative	Positive (55)
**10**	9	M	Glioblastoma Multiforme	Left Frontal lobe		Negative	Negative
**11**	12	M	Glioblastoma Multiforme	Right thalamus		55	Positive (10)
**12**	14	F	Glioblastoma Multiforme	Right temporal lobe		Negative	Negative
**13**	4	M	Glioblastoma Multiforme	Left frontal lobe		8	Negative
**14**	17	M	Glioblastoma Multiforme	Cerebellum Vermis		30	Negative
**15**	17	M	Glioblastoma Multiforme	Left temporal lobe		35	Negative
**16**	4/12	F	Glioblastoma Multiforme	Left temporal lobe		30	Positive (60)
**17**	14	F	Glioblastoma Multiforme	Pons		32	Positive (10)
**18**	16	M	Glioblastoma Multiforme	Cerebellum Vermis		30	Positive (5)

**Table 4:** Percentage distribution of molecules expression among the GBM pediatric cohort (*N* = 18).

Molecule	Grade/No./(%)
PD-L1	0 *n* = 10 (55%) 1 *n* = 5 (28%) 2 *n* =1 (5%) 3 *n* = 2 (11%)
GFAP	0 *n* = 4 (22%) 1 *n* = 14 (78%)
Olig 2	0 *n* = 14 (78%) 1 *n* = 4 (22%)
Ki 67	0 *n* = 5 (27%) 1 *n* = 2 (11%) 2 *n* = 0 (0%) 3 *n* = 11 (61%)
p53	0 *n* = 9 (50%) 1 *n* = 9 (50%)
Synaptophysin	0 *n* =17 (94%) 1 *n* = 1 (5%)

*n*—number of cases, percentage distribution (%); PD-L1 expression grading 0 none 1 weak <20% 2 moderate 20–40% 3 high >40%; GFAP expression grading 0 none 1 present; Olig 2 expression grading 0 none 1 present; Ki 67 expression grading 0 none 1 weak <10% 2 moderate 10–25% 3 >25% high; Synaptophysin expression grading 0 none 1 present; p53 expression grading 0 none 1 present.

**Table 5:** Results of statistical analysis concerning PD-L1 correlations.

Statistical Analysis
PD-L1/p53 R = 0.38 *p* < 0.05 * PD-L1/Olig2 R = 0.61 *p* < 0.01 * PD-L1/Ki 67 R = 0.36 *p* > 0.05 **

* statistical significance; ** no statistical significance.

## Data Availability

The data presented in this study are available on request from the corresponding author. The data are not publicly available due to lack of institutional online datebase.
